# A phonologically congruent sound boosts a visual target into perceptual awareness

**DOI:** 10.3389/fnint.2014.00070

**Published:** 2014-09-11

**Authors:** Ruth Adam, Uta Noppeney

**Affiliations:** ^1^Cognitive Neuroimaging Group, Max Planck Institute for Biological CyberneticsTuebingen, Germany; ^2^Department of General Psychiatry, Center of Psychosocial Medicine, University of HeidelbergHeidelberg, Germany; ^3^Institute for Stroke and Dementia Research, Ludwig-Maximilian-UniversityMunich, Germany; ^4^Department of Psychology, Centre for Computational Neuroscience and Cognitive Robotics, University of BirminghamBirmingham, UK

**Keywords:** attentional blink, audiovisual synchrony, awareness, Bayesian causal inference, crossmodal integration, multisensory integration

## Abstract

Capacity limitations of attentional resources allow only a fraction of sensory inputs to enter our awareness. Most prominently, in the attentional blink the observer often fails to detect the second of two rapidly successive targets that are presented in a sequence of distractor items. To investigate how auditory inputs enable a visual target to escape the attentional blink, this study presented the visual letter targets T1 and T2 together with phonologically congruent or incongruent spoken letter names. First, a congruent relative to an incongruent sound at T2 rendered visual T2 more visible. Second, this T2 congruency effect was amplified when the sound was congruent at T1 as indicated by a T1 congruency × T2 congruency interaction. Critically, these effects were observed both when the sounds were presented in synchrony with and prior to the visual target letters suggesting that the sounds may increase visual target identification via multiple mechanisms such as audiovisual priming or decisional interactions. Our results demonstrate that a sound around the time of T2 increases subjects' awareness of the visual target as a function of T1 and T2 congruency. Consistent with Bayesian causal inference, the brain may thus combine (1) prior congruency expectations based on T1 congruency and (2) phonological congruency cues provided by the audiovisual inputs at T2 to infer whether auditory and visual signals emanate from a common source and should hence be integrated for perceptual decisions.

## Introduction

In our natural multisensory environment, our sensory systems are exposed to a constant inflow of sensory signals. Yet, only a small subset of those signals reaches our perceptual awareness. Attentional selection has been proposed as a critical processing bottleneck that determines whether sensory signals enter our awareness (Pashler, 1984; Tombu et al., 2011). Since attentional resources are limited, allocation of attention to one stimulus may impair perception of other competing stimuli co-occurring close in time. In the laboratory, the attentional blink paradigm (Broadbent and Broadbent, 1987; Raymond et al., 1992) is a prime example illustrating limitations in attentional capacity for two rapidly successive stimuli (Chun and Potter, 1995; Marois et al., 2004; Shapiro et al., 2006; Adam et al., 2014). In an attentional blink paradigm, participants are impaired when reporting the second (T2) of two targets (T1 and T2) that are presented within a 500 ms interval amongst a rapid visual sequence of distractor items (Shapiro et al., 1997b; Dux and Marois, 2009 see Olson et al., 2001 for phonological material).

Several mechanisms have been suggested to account for the attentional blink (see Dux and Marois, 2009; Martens and Wyble, 2010 for review). Classical “bottleneck models” attribute the attentional blink to capacity limitations that prevent the second target from consolidation into working memory (Chun and Potter, 1995; Jolicoeur, 1998; Dux and Harris, 2007; Dell'acqua et al., 2009). However, explanations based on capacity limitations have recently been challenged by studies demonstrating that the attentional blink can be reduced by various factors such as (i) changing the allocation of attentional resources to T1, distracters or T2 (Nieuwenstein, 2006), or (ii) adding a distractor task to the attentional blink paradigm. In the latter case, participants showed less attentional blinks, when they were concurrently engaged in a distractor task such as free associating. The authors attributed this paradoxical pattern to a widening of participants' attention that allowed them to process T2 in addition to T1 (Olivers and Nieuwenhuis, 2005). Collectively, these studies suggest that the attentional blink may be a product of active attentional control that selectively allocates attention to target 1 and 2 and reduces attention to the distractor items (Di Lollo et al., 2005; Olivers and Nieuwenhuis, 2005; Nieuwenstein, 2006; Olivers et al., 2007).

While most previous research has focused on the visual modality, an attentional blink has also been demonstrated for auditory or tactile processing pointing toward fundamental processing limitations of the human cognitive system (Duncan et al., 1997; Arnell and Jolicoeur, 1999; Hillstrom et al., 2002; Dell'acqua et al., 2006; Shen and Mondor, 2006; Vachon and Tremblay, 2008; Horvath and Burgyan, 2011). Moreover, a so-called crossmodal attentional blink has also been observed when target 1 and target 2 were presented in different modalities suggesting that at least some processing limitations or attentional control emerge at later potentially crossmodal processing stages (Arnell and Jolicoeur, 1999; Soto-Faraco et al., 2002; Arnell and Jenkins, 2004; Ptito et al., 2008; though see Duncan et al., 1997; Potter et al., 1998; Soto-Faraco and Spence, 2002; Martens et al., 2010). Likewise, a recent EEG study showed that the auditory mismatch negativity is enhanced for trials with visual attentional blink indicating that attentional resources are shared and commonly controlled across sensory modalities (Haroush et al., 2011).

Visual attention is thought to be guided by top-down biases as well as by bottom-up stimulus salience (Desimone and Duncan, 1995; Egeth and Yantis, 1997; Buschman and Miller, 2007). It is therefore not surprising that the probability of an attentional blink depends on the salience or behavioral relevance of the second stimulus. Previous studies have shown that T2 identification rate is enhanced for physically dissimilar items (Chun and Potter, 1995; Raymond et al., 1995; Maki et al., 1997; Nieuwenstein et al., 2005), the participant's own name (Shapiro et al., 1997a) and emotional stimuli (Anderson and Phelps, 2001). A more recent study has also demonstrated that an otherwise uninformative sound presented together with T2 enables T2 to escape the attentional blink (Olivers and Van Der Burg, 2008). Importantly, an increase in T2 identification rate was observed only if the brief sound was emitted simultaneously with the second target, but not when presented 100–300 ms prior to the target. This temporal profile argues against alerting as the underlying mechanism. It suggests that the salience of the visual T2 target is amplified by a concurrent sound via genuine multisensory mechanisms that depend on audiovisual co-occurrence.

Indeed, in our multisensory world the salience of stimuli should be determined by integrating inputs from all senses. Yet, when bombarded with many different signals the brain faces the challenge to integrate only signals that are generated by a common event or object, but segregate those from different events (Roach et al., 2006). Thus, multisensory integration inherently involves solving the so-called “causal inference” problem (Welch and Warren, 1980; Shams and Beierholm, 2010). In other words, the brain needs to infer whether two sensory signals are caused by common or two different events. From a Bayesian perspective, the brain may solve this causal inference problem by combining two sorts of knowledge: (i) top-down prior knowledge and (ii) bottom-up congruency cues. First, participants have prior knowledge or expectations about whether or not two sensory signals emanate from a common source. For instance, having encountered a series of congruent audiovisual signals that were caused by a common cause participants have high expectations that future auditory and visual signals are also generated by a common event. Conversely, after incongruent audiovisual signals participants will decrease (resp. increase) their congruency (resp. incongruency) expectations. Formally, these (in)congruency expectations are referred to as common source prior. Second, participants can infer whether signals are caused by common cause from “multisensory” congruency cues that are derived from the new incoming sensory signals (i.e., the likelihood of the two signals given a common source) (Ernst and Bulthoff, 2004; Kording et al., 2007; Beierholm et al., 2009; Yu et al., 2009). The brain may use multiple cues that are abstracted from the sensory inputs at multiples levels to infer whether two signals in different modalities are generated by the same event. Most prominently, sensory signals from a common source should coincide in time and space (Wallace et al., 1996, 2004; Macaluso and Driver, 2005; Van Atteveldt et al., 2007; Lewis and Noppeney, 2010; Vroomen and Keetels, 2010; Donohue et al., 2011). Likewise, higher order congruency cues that are defined in terms of semantics or phonology (e.g., syllables) can impose important constraints on multisensory integration (Laurienti et al., 2004; Van Atteveldt et al., 2004; Noppeney et al., 2008; Adam and Noppeney, 2010).

**Figure 1:**
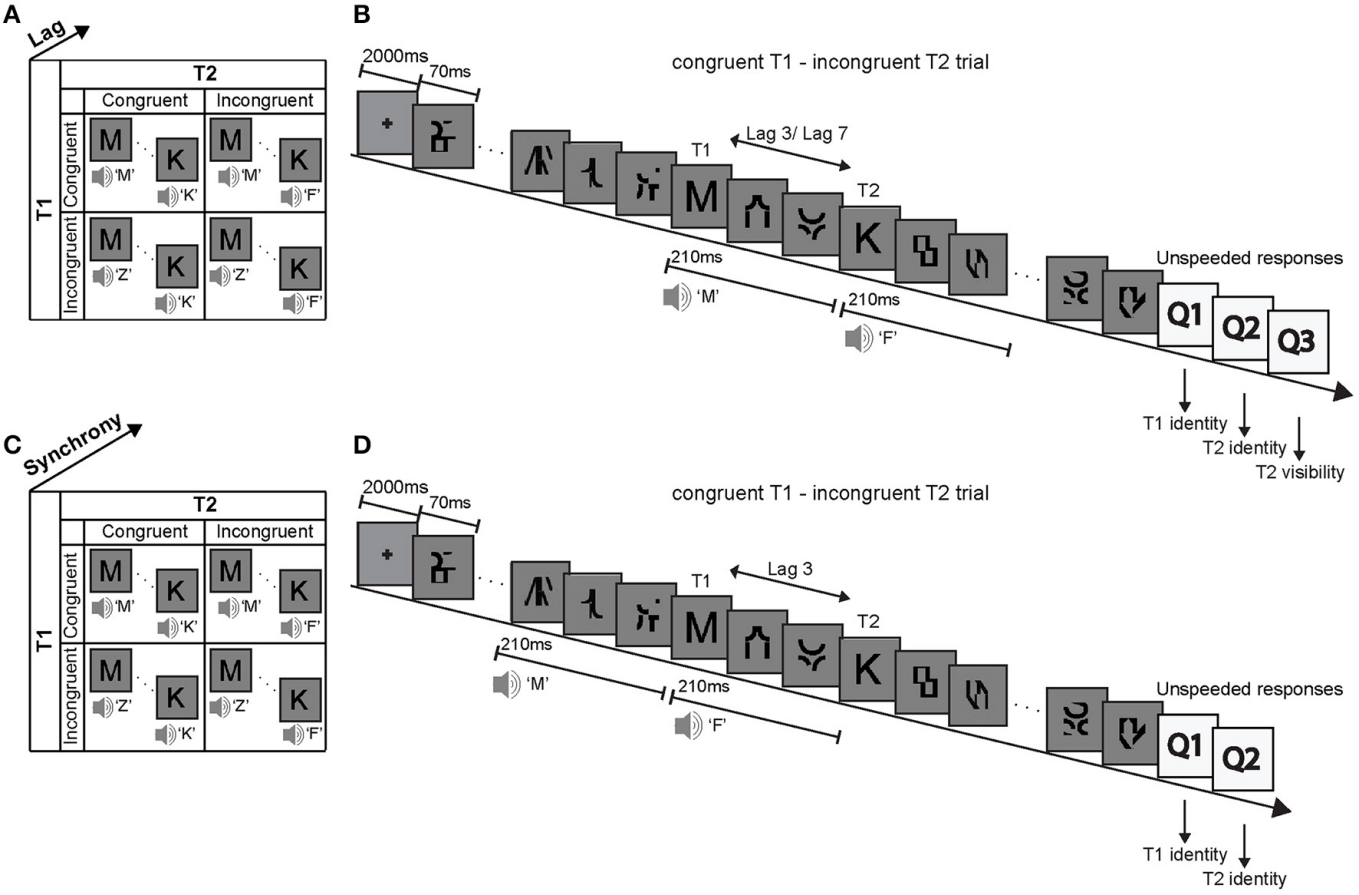
**Experimental design, example trial and stimuli**. Experiment 1: **(A)** The 2 × 2 × 2 factorial design with the factors (i) T1 AV-congruency (congruent vs. incongruent), (ii) T2 AV-congruency (congruent vs. incongruent), and (iii) lag (lag 3 vs. lag 7). **(B)** Example trial and stimuli. In an audiovisual attentional blink paradigm, participants were presented with two distinct visual target letters T1 and T2 that were accompanied by congruent or incongruent spoken letter names in a series of distractor items. Participants identified visual letter targets T1 and T2 and rated the visibility of T2. Experiment 2: **(C)** The 2 × 2 × 2 factorial design with the factors (i) T1 AV-congruency (congruent vs. incongruent), (ii) T2 AV-congruency (congruent vs. incongruent), and (iii) AV synchrony (synchrony vs. auditory-leading). **(D)** Example trial and stimuli of an auditory-leading trial. The congruent or incongruent spoken letter names were presented 210 ms before the target letters onset. T1: first target, T2: second target.

This study used a visual attentional blink paradigm to investigate how a task-irrelevant and unattended auditory signal boosts a visual signal into subjects' awareness depending on the congruency of the audiovisual (AV) signals and participants' prior congruency expectations. Specifically, in two experiments we investigated how phonologically congruent and incongruent sounds that are presented concurrently with (i.e., in synchrony) or prior to (i.e., auditory leading asynchrony) visual T1 and T2 influence subjects' T2 identification accuracy. The first experimental design factorially manipulated (1) the phonological congruency of sound 1 with T1, (2) the phonological congruency of sound 2 with T2, and (3) the lag between T1 and T2 (Figure 1A). After each trial, subjects reported the identity of T1, the identity of T2 and rated the visibility of T2 (invisible, unsure, visible). By contrast, the second experiment manipulated (1) the phonological congruency of sound 1 with T1, (2) the phonological congruency of sound 2 with T2, and (3) the synchrony between the sounds and the visual targets (Figure 1C). After each trial, subjects reported the identity of T1 and the identity of T2.

From the perspective of Bayesian causal inference, we expected an increase in T2 visibility as well as in T2 identification accuracy (i.e., a decrease in the number of attentional blinks) for phonologically congruent relative to incongruent audiovisual T2 pairs. Further, this “T2 congruency effect” should be amplified when T2 is preceded by a phonologically congruent as compared to incongruent AV T1 pair, because phonological congruency at T1 induces prior congruency expectations (i.e., a common source prior). In other words, a congruent (resp. incongruent) T1 pair will increase (resp. decrease) participant's expectations that the audiovisual signals at T2 are congruent. These prior congruency expectations will increase participants' tendency to attend to and integrate auditory and visual inputs at T2 into a unified percept resulting in an increase in accuracy for congruent trials, yet a decrease in accuracy for incongruent trials where the sound is incompatible with the visual T2 letter.

Critically, auditory, and visual signals might interact at multiple processing stages possibly implemented at different levels of the cortical hierarchy (Werner and Noppeney, 2010a,b). It is assumed that predominantly lower integration processes depend on the synchrony of the audiovisual signals, while higher order integration processes, for instance at the decisional level, are less sensitive to the precise temporal co-occurrence of the stimuli. Likewise, a prior sound may facilitate visual letter identification via crossmodal priming mechanisms that do not rely on audiovisual temporal co-occurrence (e.g., if a congruent spoken syllable precedes the visual target letter T2 identification may be facilitated).

To dissociate between mechanisms of multisensory interactions that differ in their temporal sensitivity, a follow-up experiment 2 manipulated the synchrony of the sound with respect to visual T1 and T2. If the sound and T1 or T2 are integrated into a unified percept via low level temporally sensitive mechanisms, the increase in letter identification due to congruent AV signals should depend on the synchrony of the audiovisual signals. The T2 identification accuracy should be reduced when the sound precedes T2. By contrast, we would expect a similar reduction in identification accuracy for both synchronous and asynchronous presentations when audiovisual interactions are mediated via priming or higher order decisional mechanisms.

Finally, as previously shown we expect an audiovisually incongruent T1 to reduce T2 identification accuracy (Van Der Burg et al., 2010), since audiovisual incongruent T1 pairs require greater processing demands and thereby decrease the attentional resources to be allocated to T2.

## Experiment 1

### Material and methods

#### Subjects

Thirty seven healthy subjects (20 females, mean age 26.9 years, range 18–45 years) participated in experiment 1. All subjects had normal or corrected to normal vision and reported normal hearing. Thirty five were German native speakers.

Five subjects were excluded from the analysis because they either reported themselves to be Bulgarian native speakers and were thus less familiar with German phonology (two subjects), did not complete the experiment (one subject) or they misunderstood the task and responded almost exclusively to the sound, leading to missing values in several conditions (two subjects).

Subjects gave written informed consent prior to the study as approved by the joint human research review committee of the local ethics committee of the University of Tübingen.

#### Stimuli

Visual stimuli consisted of 12 targets and 12 distractors centered on a gray background (15.4 cd/m^2^). Targets were capital Latin letters that were selected from two sets that were distinct for T1 (i.e., C, H, M, S, T, or Z) and T2 (i.e., F, J, K, N, P, or U). The letters were selected and grouped carefully according to the distinctiveness of their written letters and their spoken letter names. In addition, salient and meaningful letter combinations (e.g., T1 = P and T2 = C ⇒ PC) were avoided. Distractors were meaningless symbols created by spatially shuffling the image segments of the target letters to match the mean luminance of distractors and targets.

To decrease training effects, six stimulus sets were created, each containing the same target letters presented in a different font.

Auditory stimuli (sampling rate: 44,100 Hz, stereo, 16 bits, presented at 66 dB SPL) were the spoken German letter names corresponding to the visual target letters. Each auditory waveform was contracted to 210 ms, which left the spoken letter names fully recognizable, yet shortened their presentation time to the duration of three targets in the rapid serial visual presentation (RSVP). To avoid auditory clicks, a linear ramp of 18 ms was added to the beginning and end of the sound.

#### Design and procedure

In a visual attentional blink paradigm, subjects were presented with two visual targets (i.e., target 1: T1, target 2: T2) in a sequence of 13 rapidly presented distractor items. The visual targets were written letters selected from two non-overlapping sets of Latin letters for T1 and T2 to avoid response interference between T1 and T2 (see Stimuli section).

On each trial targets and distractors were presented at approximately 14.3 Hz (i.e., presentation duration: 70 ms, presented at visual angle 1°) in a RSVP after an initial 2000 ms fixation period (Figure 1B). T1 was presented equally often at positions 3, 4, 5, and 6. T2 was presented either 3 positions after T1 (i.e., lag 3 session) or 7 positions after T1 (i.e., lag 7 session), in separate sessions.

Concurrently with T1 and T2 onsets, a spoken letter name was presented that was phonologically congruent or incongruent to the visual target letter with an equal probability of 0.5. For instance, for congruent T1, the visual target letter “C” was presented together with the spoken letter name “Ce.” Conversely, for incongruent T1, the visual target letter “C” was presented for instance with the spoken letter name “Ha.” The auditory sound in this paradigm is exactly 50% of the time congruent and 50% of the time incongruent. Hence, if subjects responded consistently according to the sound, they would obtain 50% accuracy when averaging across all conditions. Hence, the 2 × 2 × 2 factorial design manipulated (i) T1 AV-congruency (congruent, incongruent), (ii) T2 AV-congruency (congruent, incongruent) and (3) lag between T1 and T2 (lag 3, lag 7) (Figure 1A).

In a visual selective attention paradigm, participants were instructed to attend to the visual stimuli and ignore the sounds. After each trial, subjects responded to three questions as accurately as possible in an unspeeded fashion: (1) What is the identity of T1 (C, H, M, S, T, or Z)? (2) What is the identity of T2 (F, J, K, N, P, or U)?, and (3) Rate the visibility of T2 (invisible, unsure, visible). For the identification questions, subjects were instructed to make a forced choice guess, even if they could not identify the targets. They indicated their responses on a customized keyboard. The keypress for the visibility response then triggered the next trial. Thus, our experimental paradigm combined an objective (= identification accuracy) and subjective (= visibility) criterion of observer's awareness.

Each session included 30 trials per condition amounting to 120 trials in total. Please note that all trials were of the same lag in one session, so that each session included only 4 conditions, either at lag 3 or the control condition lag 7 (Maclean and Arnell, 2012). We performed lag 3 and 7 in different sessions to make our results comparable to other studies that included only one lag, as otherwise the temporal expectancies would introduce additional variance. The order of conditions was pseudo-randomized and the letter identity was randomized with each letter appearing equally often in each condition. The assignment of lag 3 and 7 trials to separate sessions was counterbalanced. During the post-experiment inquiry, only one subject reported noticing time-differences between the two lags. In total, subjects performed nine sessions, six with lag 3 resulting in 180 trials per lag 3 condition, and three sessions with lag 7 resulting in 90 trials per lag 7 condition. This substantial number of trials was required to ensure sufficient trials per condition and visibility rating. As our study focused in particular on the lag 3 trials, we included more trials for the short T1-T2 time window (lag 3) which was our main focus. In each session, the target letters were presented in a different font to minimize learning effects that reduce the number of attentional blinks. Prior to each session, subjects were familiarized with the stimuli in the particular font setting. The familiarization procedure included four repetitions of the 12 target letters accompanied by their congruent sounds while subjects pressed the keyboard-key corresponding to the visual letter. Prior to the experiment, participants performed one practice session which included two trials per condition.

#### Apparatus

The experiment was conducted in a dimly lit experimental room. Visual stimuli were displayed on a CRT monitor (1600 × 1200 resolution, 100 Hz refresh rate, 21″ Sony CPD-G520, Japan), approximately 56 cm from the subjects' eyes. Auditory stimuli were presented at approximately 66 dB SPL, using headphones (Sennheiser HD 555MR, Germany). Experimental sessions were presented using the Cogent 2000 v1.25 (developed by the Cogent 2000 team at the FIL and the ICN and Cogent Graphics developed by John Romaya at the LON at the Wellcome Department of Imaging Neuroscience, UCL, London, UK; http://www.vislab.ucl.ac.uk/cogent.php) running under MATLAB (Mathworks Inc., Natick, MA, USA) on a Windows PC.

#### Data analysis

Operationally, awareness was defined based on subjects' report at the end of the trial. In experiment 1, we employed two different reports: visual letter identification and visibility judgment. Data analysis was limited to trials where subjects correctly identified the T1 letter. In other words, all measures were contingent on T1 correctness.

We assessed observer's awareness of the T2 using two criteria (following recommendation by Dehaene and Changeux, 2011). First, in accordance with most attentional blink studies, we employed subjects' visual letter identification accuracy at T2 as an objective index of visual awareness. Critically, visual letter identification at T2 was limited to only those trials where participants correctly identified T1 (i.e., % correct T2 identification contingent on correct T1 identification: %T2|T1). Second, we used subjects' visibility judgment (i.e., the percentage judged visible) as a subjective criterion again limited to only those trials where T1 was correctly identified (Sergent and Dehaene, 2004; Nieuwenhuis and De Kleijn, 2011). The objective index is thought to be independent of subjects' response criterion, yet may overestimate visual awareness, because subjects can perform better than chance even for stimuli they are not aware of (e.g., correct responses in blindsight; Weiskrantz et al., 1974; Persaud and Lau, 2008). Conversely, the subjective index depends on where subjects set their internal visibility criterion, yet may be more inclusive.

### Results and discussion

The overall mean T1 identification accuracy (±s.e.m.) was 82.7 ± 2.3%. A 2 × 2 repeated measures ANOVA of % T1 identification accuracy with the within subject factors lag (3 vs. 7) and T1 AV-congruency (congruent vs. incongruent) revealed a T1 congruency main effect on T1 performance [*F*_(1, 31)_ = 25.42, *p* < 0.001, partial η^2^ = 0.451], with reduced accuracy for incongruent (77.0 ± 3.0%) relative to congruent (88.4 ± 2.0%) AV pairs. No other effects were significant.

#### Objective awareness criterion: T2 identification accuracy (given T1 is correct)

The 2 (lag: 3 vs. 7) × 2 (T1 congruency: congruent vs. incongruent) × 2 (T2 congruency: congruent vs. incongruent) repeated measures ANOVA of % T2 identification accuracy (given correct identification of T1) revealed main effects of lag, T1 congruency and T2 congruency. Consistent with the well-established timecourse of the attentional blink, T2 accuracy was increased for lag 7 relative to lag 3 validating our attentional blink paradigm (Raymond et al., 1992). Nevertheless, identification accuracy was still reduced even for lag 7 trials, potentially because the audiovisual T1 pairs (especially the incongruent target-sound pairs, Van Der Burg et al., 2010) are more difficult to process than the standard purely visual T1 thereby protracting the attentional blink. Further, T2 identification accuracy decreased both for incongruent T1 and incongruent T2 pairs as indicated by the two congruency main effects. In other words, fewer attentional blinks were observed when the auditory sound matched T2 (79.8 ± 2.5% for congruent vs. 67.2 ± 3.1% for incongruent T2 pair) (see Table 1). Yet, these main effects need to be interpreted with caution as we also observed a 3 way interaction (see below).

**Table 1:** **Statistical results of experiment 1**.

**Factor**	**Objective reports**	**Subjective reports**
**Statistical results from the three-way ANOVAs (*df*: 1,31)**		
Lag	*F* = 28.24, *p* < 0.001^*^ partial η^2^ = 0.477	*F* = 15.38, *p* < 0.001^*^ partial η^2^ = 0.332
T1 congruency	*F* = 34.85, *p* < 0.001^*^ partial η^2^ = 0.529	*F* = 38.57, *p* < 0.001^*^ partial η^2^ = 0.554
T2 congruency	*F* = 35.61, *p* < 0.001^*^ partial η^2^ = 0.535	*F* = 18.15, *p* < 0.001^*^ partial η^2^ = 0.369
T1 congruency × lag	*F* = 1.41, *p* = 0.244 partial η^2^ = 0.044	*F* = 0.001, *p* = 0.977 partial η^2^ < 0.001
T2 congruency × lag	*F* = 6.37, *p* = 0.017^*^ partial η^2^ = 0.171	*F* = 0.48, *p* = 0.493 partial η^2^ = 0.015
T1 congruency × T2 congruency	*F* = 2.92, *p* = 0.097 partial η^2^ = 0.086	*F* = 6.14, *p* = 0.019^*^ partial η^2^ = 0.165
T1 congruency × T2 congruency × lag	*F* = 6.42, *p* = 0.017^*^ Partial η^2^ = 0.172	*F* = 0.64, *p* = 0.430 partial η^2^ = 0.020
**Mean ± s.e.m. identification accuracy and visibility judgment (given T1 correct) in the 8 conditions**		
T1 congruent & T2 congruent & lag 3	0.80 ± 0.03	0.49 ± 0.05
T1 congruent & T2 incongruent & lag 3	0.62 ± 0.04	0.42 ± 0.04
T1 incongruent & T2 congruent & lag 3	0.73 ± 0.03	0.43 ± 0.05
T1 incongruent & T2 incongruent & lag 3	0.62 ± 0.03	0.38 ± 0.05
T1 congruent & T2 congruent & lag 7	0.86 ± 0.02	0.57 ± 0.05
T1 congruent & T2 incongruent & lag 7	0.75 ± 0.03	0.51 ± 0.04
T1 incongruent & T2 congruent & lag 7	0.81 ± 0.03	0.50 ± 0.05
T1 incongruent & T2 incongruent & lag 7	0.70 ± 0.03	0.47 ± 0.04

* p < 0.05.

**Figure 2:**
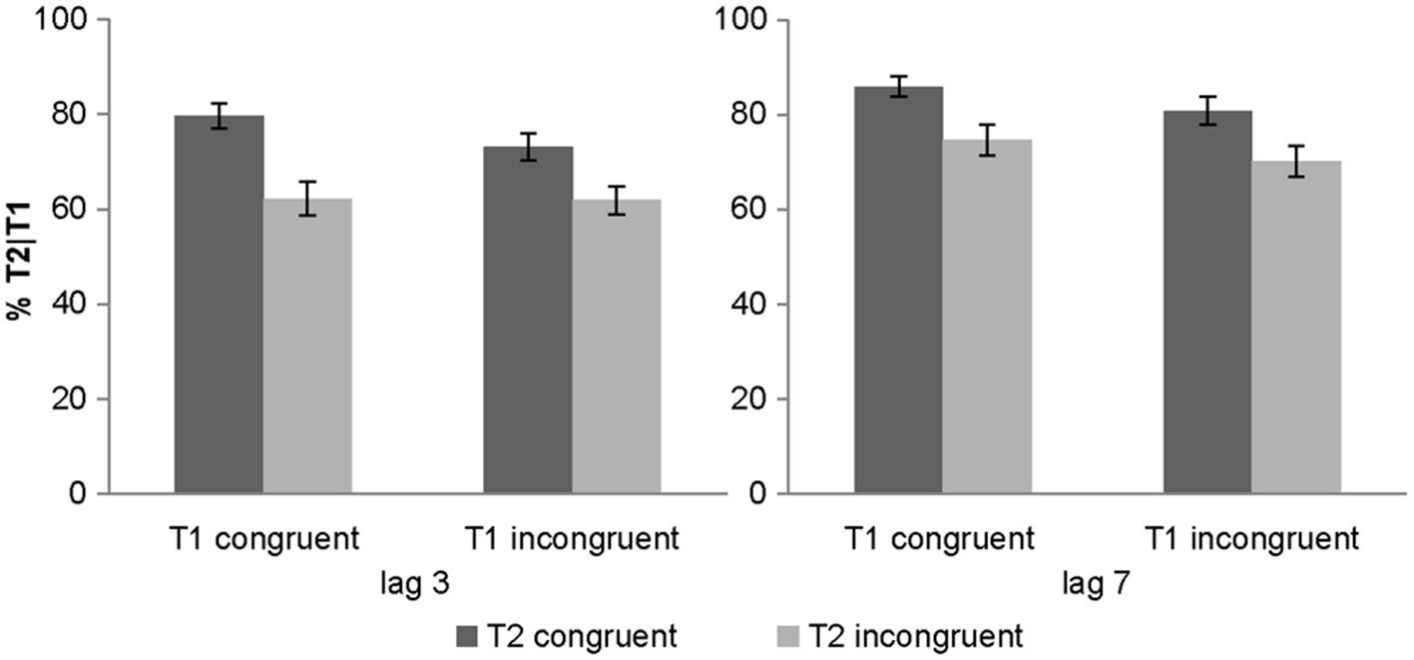
**Objective awareness criterion in experiment 1**. T2 identification accuracy (% T2 correct conditional on T1 correct) (across subjects' mean ± s.e.m.) for the 8 different conditions.

We also observed a significant 2-way interaction between lag x T2 congruency with greater T2 congruency effects for lag 3 vs. lag 7 [*post-hoc t*-test for lag 3: *t*_(31)_ = 6.01, *p* < 0.001, mean difference = 14.3%; *post-hoc t*-test for lag 7: *t*_(31)_ = 5.35, *p* < 0.001, mean difference = 10.9%]. Critically, there was a trend for T1 congruency × T2 congruency interaction and in particular a significant 3-way interaction. To further evaluate this 3-way interaction, we tested for the T1 congruency × T2 congruency effects separately for the two lags. These additional ANOVAs revealed a significant T1 × T2 interaction only for lag 3 [*F*_(1, 31)_ = 6.84, *p* = 0.014, partial η^2^ = 0.181], but not for lag 7 [*F*_(1, 31)_ = 0.1, *p* = 0.755, partial η^2^ = 0.003]. Follow up *post-hoc t*-tests on the interaction at lag 3 showed significant but stronger T2 congruency effects when T1 is congruent [*t*_(31)_ = 5.13, *p* < 0.001, mean difference = 17.3%] relative to when it is incongruent [*t*_(31)_ = 6.98, *p* < 0.001, mean difference = 11.2%]. These results demonstrate that the audiovisual T2 congruency effect is amplified for audiovisually congruent T1 pairs at lag 3 (Figure 2). This T1 × T2 interaction at lag 3 was hypothesized based on models of Bayesian causal inference. Basically, as participants have some tendency to integrate audiovisual signals that are close in time and space, we observe higher identification accuracy when the auditory signal provide congruent (i.e., facilitatory) relative to incongruent (i.e., interfering) information. Importantly, if T1 is congruent and participants expect T2 audiovisual signals to be congruent, audiovisual integration will be amplified at T2 leading to enhanced audiovisual T2 congruency effects.

Critically, the interpretation of this interaction remains to some extent ambiguous, as our experimental paradigm did not include any “neutral” audiovisual condition that is neither congruent nor incongruent. In fact, we would argue that a truly neutral condition does not exist. One may suggest a unisensory condition without any auditory T2 may be included as a neutral condition. However, a previous study demonstrated that even a simple beep changes the attentional processing at T2 (Olivers and Van Der Burg, 2008). Likewise, a “beep” is not an ideal “neutral” control condition, as it differs in sound complexity and cognitive processing demands from the spoken syllables. Hence, it seems difficult or even impossible to generate a neutral condition that is neither congruent nor incongruent and yet tightly matched to the spoken syllables in terms of processing demands (e.g., phonemic recognition etc.). The absence of a neutral condition makes the interpretation of participant's response profile ambiguous.

At first sight, the accuracy profile for lag 3 conditions in Figure 2 may suggest that T1 congruency increases the accuracy on T2 congruent trials without reducing the accuracy on T2 incongruent trials. In other words, T1 congruency only facilitates identification of congruent T2 without inducing interference for incongruent T2 trials. This would be a surprising finding because from the perspective of Bayesian causal inference, we would expect T1 congruency to increase participants' congruency expectations and hence their tendency to integrate audiovisual signals at T2 irrespective of T2 congruency. Enhanced audiovisual integration at T2 should then lead to both an increase in accuracy for congruent T2 pairs (= AV facilitation) and a decrease in accuracy for incongruent T2 pairs (= AV interference).

Yet, we may also explain this response profile by assuming that incongruent T1 pairs exert two distinct effects. First, as previously suggested, incongruent T1 should place more demands on processing and therefore generally decrease T2 accuracy for both congruent and incongruent T2 signals (Van Der Burg et al., 2010). Second, as described above incongruent T1 signals should also make subjects less likely to integrate AV signals at T2 again regardless of their congruency. This second mechanisms should then lead to a decrease in accuracy for congruent T2 signals and an increase in accuracy for incongruent T2 signals (by reducing the interference from the incongruent auditory signal at T2). Thus, T1 (in)congruency would have opposite effects on processing incongruent T2 signals via those to mechanisms; yet, T1 (in)congruency would have the same effect on congruent T2 signals. Assuming that T1 (in)congruency influences T2 processing concurrently via both mechanisms, the T1 (in)congruency effect on incongruent T2 signals may be canceled out.

In conclusion, a combination of a general main effect of T1 (in)congruency (i.e., incongruent relative to congruent T1 signals decrease accuracy for both T2 congruent and incongruent trials) and an interaction between T1 × T2 congruency (i.e., incongruent relative to congruent T1 signals decrease accuracy for congruent T2 and increase accuracy for incongruent T2 trials) may then induce an accuracy profile where T1 congruency apparently leads only to a facilitation for congruent T2, but no interference for incongruent T2 trials (i.e., no decrease in accuracy for incongruent relative to congruent T1 on incongruent T2 trials).

To further investigate whether T1 congruency influences the audiovisual binding of incongruent T2 pairs, we therefore analyzed subjects' error responses on T2 incongruent trials. The basic hypothesis was that if audiovisual T1 congruency induces a congruency prior that generally increases the binding of audiovisual signals at T2, subjects should more frequently misidentify T2 according to the spoken letter name, when T1 is congruent relative to incongruent.

Hence, we computed the fraction of T2 incongruent trials where subjects reported the identity of the spoken letter name rather than an unrelated letter name. A 2 (lag: 3 vs. 7) × 2 (T1 congruency: congruent vs. incongruent) repeated measures ANOVA on the fraction of trials in which the spoken letter name was reported out of all incorrect trials revealed a significant main effect of T1 congruency (Table 2). More specifically, the identity of the spoken letter name was more frequently reported when the trial started with a congruent T1 (42.6 ± 3.6%) relative to an incongruent T1 (36.3 ± 2.4%). This is in line with the prediction of Bayesian causal inference where prior congruency expectations will increase audiovisual interference if the two signals are incongruent.

**Table 2:** **Reports according to sound in experiment 1: statistical results from the Two-Way ANOVA**.

**Factor (*df*: 1, 31)**	
Lag	*F* = 0.04, *p* = 0.841 partial η^2^ = 0.001
T1 congruency	*F* = 5.64, *p* = 0.024^*^ partial η^2^ = 0.154
T1 congruency × Lag	*F* = 0.63, *p* = 0.433 partial η^2^ = 0.020

* p < 0.05.

#### Subjective awareness criterion: visibility judgment (given T1 correct)

**Figure 3:**
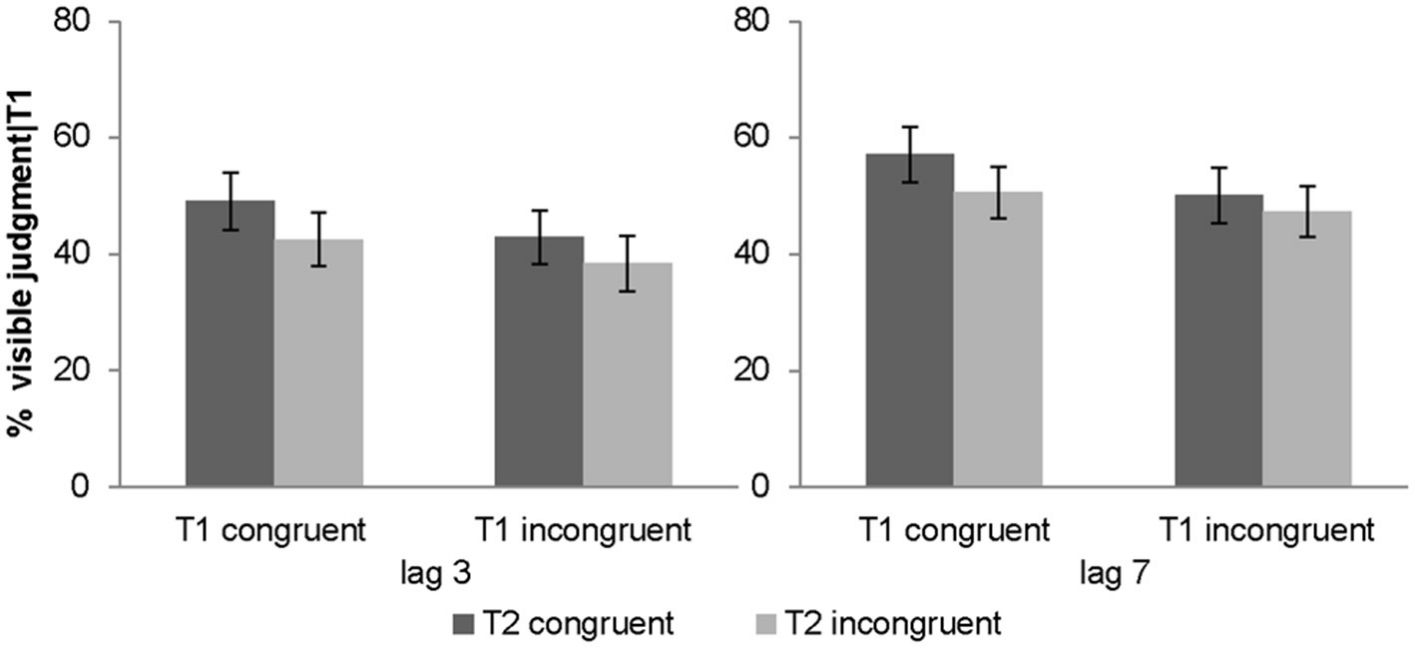
**Subjective awareness criterion in experiment 1 (visibility judgment)**. Percentage of visible targets given T1 correct (across subjects' mean ± s.e.m.) for the 8 different conditions.

Percentage of T2 targets judged visibly was used as a complementary subjective measure of awareness. The 2 (lag: 3 vs. 7) × 2 (T1 congruency: congruent vs. incongruent) × 2 (T2 congruency: congruent vs. incongruent) repeated measures ANOVA of % judged visible revealed a significant main effect of T1 congruency, T2 congruency and lag. T2 visibility was increased for congruent T1, congruent T2 and lag 7 (see Table 1). Furthermore, there was a significant interaction between T1 and T2 congruency. Follow up *post-hoc t*-tests on the T2 congruency effects for visibility judgments showed significant but stronger T2 congruency effects when T1 is congruent [*t*_(31)_ = 4.01, *p* < 0.001, mean difference = 6.5%] relative to when it is incongruent [*t*_(31)_ = 3.88, *p* = 0.001, mean difference = 3.6%]. In other words, T2 target visibility was enhanced for congruent relative to incongruent T2 pairs, and this T2 congruency effect was enhanced by congruent T1 pairs (Figure 3). Importantly, even though the objective and subjective awareness indices showed some small differences in results pattern (e.g., 3-way interaction only for objective index), they both converged in showing an interaction between T1 and T2 congruency at least for short lag as expected under Bayesian causal inference.

## Experiment 2

### Material and methods

The second experiment investigated whether the congruency effects that we observed in the first experiment for lag 3 were dependent on audiovisual synchrony. Thus, the experimental paradigm was basically identical to the first experiment apart from the following modifications:

#### Subjects

16 healthy subjects participated in the second experiment (11 females, mean age 25.1 years, range 19–30 years). As experiment 2 was partly a replication of experiment 1 and we could therefore use directed tests based on strong a priori hypotheses, we included fewer subjects in this experiment. One subject was excluded due to problems with the setup, resulting 15 subjects in the final analysis. All subjects were German native speakers, had normal or corrected to normal vision and reported normal hearing.

#### Design and procedure

The 2 × 2 × 2 factorial design manipulated (i) T1 AV-congruency (congruent, incongruent), (ii) T2 AV-congruency (congruent, incongruent), and (iii) AV synchrony (synchronous, auditory-leading) (Figure 1C).

In a visual attentional blink paradigm, subjects were presented with T1 and T2 embedded in a sequence of 13 rapidly presented distractor items. T1 was presented equally often at positions 5, 6, 7, and 8. In this way we avoided presenting the sounds in synchrony with distractor one in the asynchronous auditory-leading case. T2 was always presented at lag 3 where most attentional blinks occur. As in experiment 1, a spoken letter name was played together with T1 and T2 onset in synchronous trials. In the auditory-leading condition, the sound onset was 210 ms prior to the target presentation. Thus, in auditory-leading trials, the T1 sound onset was synchronous with a distractor and the T2 sound onset was synchronous with the presentation of visual T1 (Figure 1D). If the effect of the sounds on visual identification is strictly dependent on audiovisual synchrony, the presentation of the 2nd sound in synchrony with T1 should induce an incongruency effect irrespective of T2 congruency. Hence, the observation of a T1 × T2 congruency interaction despite this design choice would point toward neural mechanisms that do not strictly depend on audiovisual synchrony. However, the effect of the spoken T2 syllables on T1 identification may be minimal, because T1 and T2 were selected two distinct stimulus sets.

As the subjective and objective indices of awareness provided basically equivalent results in experiment 1, experiment 2 focused only on the objective awareness index that is traditionally used in attentional blink paradigms. Thus, after each trial, participants were asked only to report: (1) What is the identity of T1? (2) What is the identity of T2?

Subjects performed four sessions for synchronous and four sessions for asynchronous audiovisual presentations amounting to 120 trials per condition. Audiovisual synchrony was manipulated across sessions in order to control for temporal expectancies and make the results comparable across our two experiments. The order of the audiovisual synchrony sessions was pseudo-randomized. Prior to the experiment, participants performed one practice session which included two trials per condition.

#### Apparatus

The experiment was conducted in a dimly lit cubicle. Visual stimuli were displayed on a LCD monitor (1600 × 12000 resolution, 60 Hz refresh rate, 20.1″, DELL 2007FP, US), placed approximately 56 cm from the subjects' eyes.

### Results and discussion

The overall mean T1 identification accuracy was 82.04 ± 3.7%.A 2 × 2 repeated measures ANOVA of % T1 identification accuracy with the factors AV synchrony (synchronous vs. auditory-leading) and T1 AV-congruency (congruent vs. incongruent) revealed a main effect of T1 congruency [*F*_(1, 14)_ = 8.03, *p* = 0.013, partial η^2^ = 0.365], with deceased accuracy for incongruent relative to congruent stimuli (88.5 ± 4.0% for congruent and, 75.6 ± 4.7% accuracy for incongruent T1). No other effects were significant.

#### Objective awareness criterion: T2 identification accuracy (given T1 is correct)

The 2 (AV synchrony: synchronous vs. auditory-leading) × 2 (T1 congruency: congruent vs. incongruent) × 2 (T2 congruency: congruent vs. incongruent) repeated measures ANOVA of % T2 accuracy indicated a significant main effect of T2 congruency and a trend for main effect of T1 congruency (*p* = 0.065). In line with experiment 1, T2 identification accuracy decreased for incongruent T2 pairs (78.6 ± 4.2%, 57.1 ± 5.8% accuracy for congruent and incongruent T2, respectively) (see Table 3).

**Table 3:** **Statistical results of experiment 2**.

**Factor**	**Objective reports**
**Statistical results from the three-way ANOVA (*df*: 1,14)**	
Synchrony	*F* = 0.19, *p* = 0.669 partial η^2^ = 0.013
T1 congruency	*F* = 4.00, *p* = 0.065^∧^ partial η^2^ = 0.222
T2 congruency	*F* = 16.16, *p* = 0.001^*^ partial η^2^ = 0.536
T1 congruency × synchrony	*F* < 0.1, *p* = 1.000 partial η^2^ = 0.00
T2 congruency × synchrony	*F* = 0.31, *p* = 0.587 partial η^2^ = 0.022
T1 congruency × T2 congruency	*F* = 4.23, *p* = 0.059^∧^ partial η^2^ = 0. 232
T1 congruency × T2 congruency × synchrony	*F* = 0.55, *p* = 0.819 partial η^2^ = 0.004
**Mean ± s.e.m. identification accuracy (given T1 correct) in the 8 conditions**	
T1 congruent & T2 congruent & synchronous	0.82 ± 0.04
T1 congruent & T2 incongruent & synchronous	0.57 ± 0.06
T1 incongruent & T2 congruent & synchronous	0.75 ± 0.05
T1 incongruent & T2 incongruent & synchronous	0.59 ± 0.05
T1 congruent & T2 congruent & auditory-leading	0.82 ± 0.04
T1 congruent & T2 incongruent & auditory-leading	0.55 ± 0.07
T1 incongruent & T2 congruent & auditory-leading	0.75 ± 0.05
T1 incongruent & T2 incongruent & auditory-leading	0.57 ± 0.06

* p < 0.05,

∧ p < 0.10.

**Figure 4:**
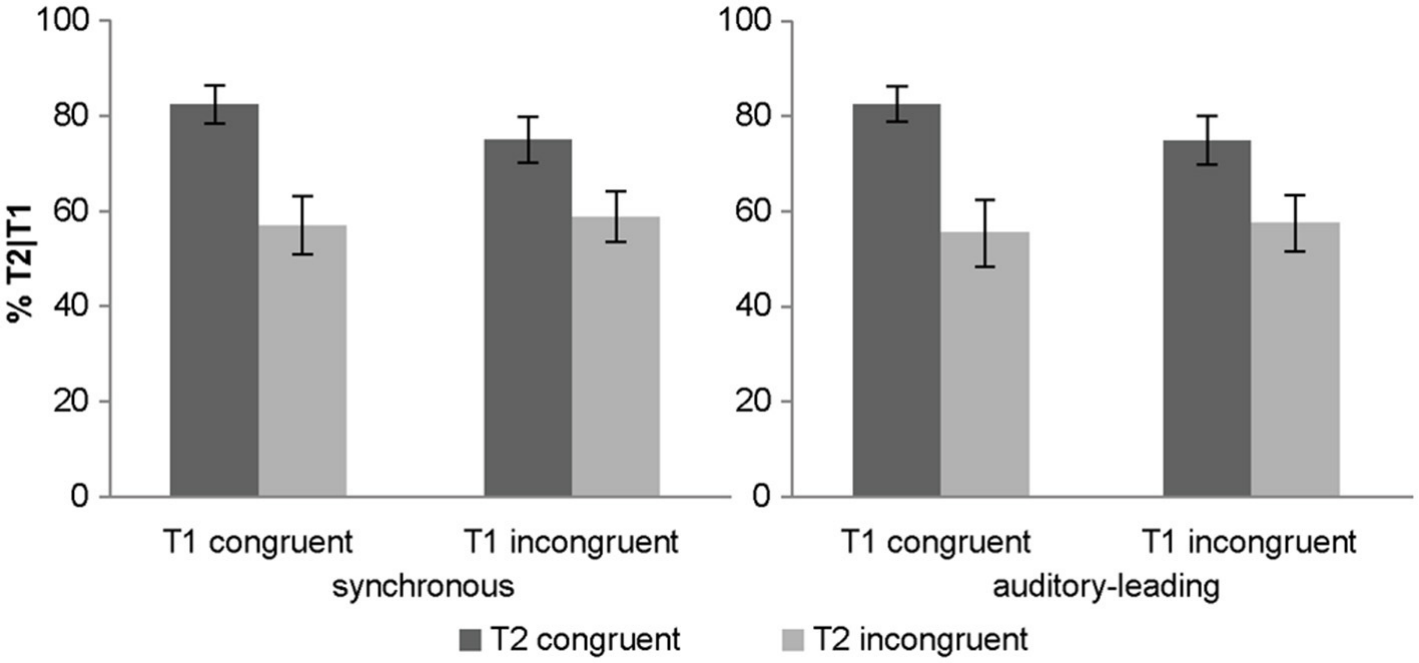
**Objective awareness criterion in experiment 2**. T2 identification accuracy (% T2 correct conditional on T1 correct) (across subjects' mean ± s.e.m.) for the 8 different conditions.

Importantly, there was a trend for a two way T1 congruency × T2 congruency interaction (*p* = 0.059). Experiment 1 demonstrated an interaction between T1 × T2 congruency which serves as a directed a priori hypothesis for experiment 2. Hence, based on this a priori hypothesis, we could test for a directed interaction resulting in a *p*-value = 0.03. As in experiment 1, T1 congruency amplified the congruency effect of T2 for both synchronous and asynchronous conditions (Figure 4). *Post-hoc t*-tests on the T2 congruency effects showed significant but stronger T2 congruency effects when T1 is congruent [*t*_(14)_ = 4.05, *p* < 0.001, mean difference = 26.3%] relative to when it is incongruent [*t*_(14)_ = 3.31, *p* = 0.005, mean difference = 16.8%].

In summary, experiment 2 replicated the effects we observed in experiment 1 for both synchronous and asynchronous (i.e., auditory-leading) conditions. The slightly less significant effects are most likely due to smaller number of subjects included in experiment 2. Note, however, that the magnitude of the difference between the congruent and the incongruent conditions was larger compared to the one observed in experiment 1. Importantly, we did not observe any interactions between synchrony and T1 or T2 congruency indicating that the congruency effects do not always rely critically on the synchrony of the audiovisual signals. Collectively, these results suggest that a sound can boost the visual target into awareness also via mechanisms that do not critically depend on audiovisual timing (e.g., audiovisual priming in the asynchronous condition or interactions at the decisional level).

## General discussion

In our natural environment our senses are constantly bombarded by many different signals with only a small fraction of them entering our awareness (Raymond et al., 1992; Simons and Chabris, 1999; Sergent et al., 2005; Pourtois et al., 2006). This study investigated how the brain selects visual signals for conscious perception. Specifically, we examined whether the awareness of visual signals is influenced by auditory signals. Using the attentional blink paradigm, we demonstrate that spoken syllables boost visual letters into subjects' awareness depending on audiovisual congruency and subjects' prior congruency expectations. As the audiovisual congruency effects did not always rely critically on audiovisual synchrony, they may be mediated potentially via multiple mechanisms such as audiovisual binding, crossmodal priming or even interference/facilitation at the decisional level.

Our results suggest that audiovisual interactions play a critical role in shaping visual awareness as measured by participants' accuracy in the letter identification task and subjective visibility judgments. Previous research into perceptual awareness has focused primarily on signals from one sensory domain. Most prominently, visual, auditory and tactile signals were shown to evade conscious perception when presented in a rapid stream of distractor items (Sergent and Dehaene, 2004; Dell'acqua et al., 2006; Horvath and Burgyan, 2011). Yet, the question whether sensory signals are selected for awareness independently for each sensory modality or interactively across the senses remains open (see related research on multistability and rivalry in a multisensory context: van Ee et al., 2009; Conrad et al., 2010, 2012, 2013; Lunghi et al., 2010, 2014). In the latter case, auditory signals may influence subjects' visual awareness via several multisensory mechanisms.

To investigate whether and how auditory signals modulate subjects' visual awareness, we presented the written T1 and T2 letters together with spoken letter names in an attentional blink paradigm (Raymond et al., 1992). The spoken letter names were either congruent or incongruent with respect to the written T1 and T2 letters. As congruent and incongruent spoken letter names were presented with equal probability, subjects that relied solely on the spoken letter names for making their decision should obtain 50% accuracy averaged across all conditions. In the following, we will first discuss the main effects of T1 and T2 congruency on identification accuracy and then the critical interaction between T1 and T2 congruency within the framework of Bayesian Causal Inference.

First, we demonstrate that incongruent T1 pairs decreased both T1 identification accuracy and T2 identification accuracy in particular for congruent audiovisual T2 signals (for related findings see Van Der Burg et al., 2010). Thus, audiovisually incongruent T1 pairs place greater processing demands at T1 and thereby reduce the attentional resources available for T2 processing resulting in decreased performance (Visser, 2007; Giesbrecht et al., 2009; Burt et al., 2011).

Second and more importantly, we investigated the effect of audiovisual congruency at T2 on visual awareness. From the perspective of Bayesian causal inference, audiovisual congruency is an important cue informing the brain whether visual and auditory signals are generated by a common source and should hence be combined for a perceptual decision or even integrated into a unified percept (Roach et al., 2006; Shams and Seitz, 2008). Hence, we expected audiovisual congruency at T2 to facilitate audiovisual processing, which in turn should enable recognition of visual signals. Indeed, subjects were more likely to report the correct written T2 letter, when it was presented together with a congruent spoken letter name. Convergent results were provided by the subjective criterion of awareness, i.e. the visibility judgment of T2 letter. Critically, this subjective criterion of awareness showed the same profile across conditions with an increase in visibility for audiovisually congruent relative to incongruent T2. This increase in stimulus perceptibility for congruent relative to incongruent T2 targets suggests that auditory signals influence visual awareness. Next, we investigated whether audiovisual facilitation relies strictly on audiovisual synchrony as would be expected for low level automatic integration processes. Yet in contrast to this conjecture, experiment 2 demonstrated that a prior sound that preceded the visual target by 210 ms induced a similar increase in letter identification. These results suggest that the facilitation of T2 identification in the attentional blink paradigm does not necessitate time-sensitive audiovisual integration mechanisms. Instead, several mechanisms may be involved in mediating the facilitation induced by a prior congruent relative to an incongruent sound. Most prominently, a prior congruent sound (e.g., in the context of asynchronous presentation) may facilitate T2 identification via mechanisms of audiovisual (i.e., crossmodal) priming. Alternatively, auditory and visual signals may interact at higher processing levels that are less constrained by temporal co-occurrence.

In the next step, we examined whether audiovisual congruencies at T1 and T2 interact as predicted by Bayesian causal inference where a top-down congruency prior is combined with bottom-up congruency cues derived from new sensory signals to infer whether two sensory signals should be integrated. Indeed, a congruent T1 pair amplified the increase in visibility and performance accuracy for congruent relative to incongruent T2 pairs both for synchronous and auditory-leading presentation.

Conversely, subjects responded more frequently according to the spoken letter name, when incongruent T2 pairs were preceded by a congruent T1 pair. In other words, subjects' response was more strongly influenced by the incongruent auditory letter name in trials that started with a congruent T1. Thus, in line with Bayesian causal inference, a congruent T1 pair induces observers to form a congruency prior, i.e., the prior expectation that subsequent auditory and visual signals pertain to the same event and should hence be integrated. The congruency expectations then in turn enhance audiovisual interactions at T2 leading to greater benefits for congruent T2 pairs (facilitation) and/or audiovisual interference for incongruent T2 pairs. As our study did not include any neutral condition, these two aspects (i.e., interference for incongruent or facilitation for congruent audiovisual signals) cannot be distinguished. Collectively, our results suggest that participants combine prior congruency expectations (formed on the basis of T1) with incoming phonological congruency cues (provided by T2) to determine whether auditory and visual signals should be combined for perceptual decisions. In the congruent case, audiovisual interactions boost visual signals into awareness leading to higher identification accuracy and visibility. Conversely, in the incongruent case, they lead to audiovisual interference. Importantly, these audiovisual congruency effects were observed for both audiovisual synchronous and auditory-leading presentations suggesting that the audiovisual interactions emerge potentially via several mechanisms at least some of which do not critically rely on temporal synchrony such as crossmodal priming in the asynchronous conditions.

Yet, as a cautionary note we should add that awareness in this and many other paradigms is operationally defined based on whether or not participants are able to correctly report T2 letter identity at the end of the trial. Hence, as an alternative explanatory mechanism audiovisual integration may not facilitate awareness *per se*, but stabilize memory representations such that they are more reportable at the end of the trial. This alternative mechanism may be further investigated in paradigms that also manipulate the delay between audiovisual stimulation and report of target identity.

Collectively, our results demonstrate that audiovisual interactions may affect perceptual awareness in attentional blink paradigms at multiple levels. First, audiovisual integration or priming (in the asynchronous case) mechanisms (Soto-Faraco et al., 2004; Lewis and Noppeney, 2010; Talsma et al., 2010; Werner and Noppeney, 2010a) may boost the bottom-up salience of the visual stimulus thereby facilitating perceptual awareness. As awareness in the attentional blink paradigm is closely related to attentional selection, some of these mechanisms may act preattentively. Second, audiovisual interactions may influence perceptual decision mechanisms as previously described in audiovisual congruency manipulations (Adam and Noppeney, 2010; Conrad et al., 2010; Noppeney et al., 2010; Werner and Noppeney, 2010a; Hsiao et al., 2012), Stroop (Banich et al., 2000; MacDonald et al., 2000; Kane and Engle, 2003; Egner and Hirsch, 2005; Egner, 2007) and flanker (Gratton et al., 1992; Botvinick et al., 1999; Lavie et al., 2003; Egner, 2007; Yu et al., 2009) tasks. Audiovisual interactions at all stages ranging from audiovisual integration or priming in the absence of awareness to decisional processes may be governed by Bayesian causal inference (Kording et al., 2007; Yu et al., 2009) as normative computational principles that enable optimal perception of the environment. Bayesian causal inference normatively describes the computational principles that the brain should use to determine whether or not to combine information from multiple sources in processes that range from low level automatic audiovisual interactions to higher order perceptual decisions. The brain may determine whether sensory signals should interact or be segregated by combining prior congruency information (based on T1) and incoming sensory evidence (T2).

Future neuroimaging studies (e.g., fMRI, EEG, MEG) are needed to track and dissociate the neural processes underlying multisensory interactions at multiple levels of the processing hierarchy throughout unaware and aware processing stages. For instance, prior congruency expectations may affect multisensory integration through modulatory activity in the left prefrontal cortex that has previously been implicated in cognitive control (Kerns et al., 2004; Rushworth et al., 2004; Brown and Braver, 2005; Carter and Van Veen, 2007; Orr and Weissman, 2009). Thus, in the Stroop color-naming task (naming the ink-color of a color word), prior incongruent trials increased inferior frontal sulcus (IFS) activation and top-down modulation which in turn reduced interference from irrelevant and incongruent information on subsequent trials (Kerns et al., 2004). Conversely, different types of incongruency relationships may be processed at distinct levels of the cortical hierarchy including temporal congruency at the primary cortical level (e.g., Noesselt et al., 2007; Lewis and Noppeney, 2010; Lee and Noppeney, 2014) and phonological or semantic congruency at higher order association areas (Ojanen et al., 2005; Pekkola et al., 2006; Von Kriegstein and Giraud, 2006; Hein et al., 2007; Van Atteveldt et al., 2007; Adam and Noppeney, 2010; Yoncheva et al., 2010).

### Conflict of interest statement

The authors declare that the research was conducted in the absence of any commercial or financial relationships that could be construed as a potential conflict of interest.
